# Cross-Reacting Antibacterial Auto-Antibodies Are Produced within Coronary Atherosclerotic Plaques of Acute Coronary Syndrome Patients

**DOI:** 10.1371/journal.pone.0042283

**Published:** 2012-08-06

**Authors:** Filippo Canducci, Diego Saita, Chiara Foglieni, Maria Rosaria Piscopiello, Roberto Chiesa, Antonio Colombo, Domenico Cianflone, Attilio Maseri, Massimo Clementi, Roberto Burioni

**Affiliations:** 1 Laboratory of Microbiology and Virology, Ospedale San Raffaele, Milan, Italy; 2 University Vita-Salute San Raffaele, Milan, Italy; 3 Clinical Cardiovascular Biology Centre, Ospedale San Raffaele, Milan, Italy; 4 Istituto di Neurologia Sperimentale, Ospedale San Raffaele, Milan, Italy; 5 Division of Vascular Surgery, Ospedale San Raffaele, Milan, Italy; 6 Interventional Cardiology Unit, Department of Cardio-Thoracic and Vascular Diseases, Ospedale San Raffaele, Milan Italy; 7 Heart Care Foundation, ONLUS, Florence, Italy; University of Udine, Italy

## Abstract

Coronary atherosclerosis, the main condition predisposing to acute myocardial infarction, has an inflammatory component caused by stimuli that are yet unknown. We molecularly investigated the nature of the immune response within human coronary lesion in four coronary plaques obtained by endoluminal atherectomy from four patients. We constructed phage-display libraries containing the IgG1/kappa antibody fragments produced by B-lymphocytes present in each plaque. By immunoaffinity, we selected from these libraries a monoclonal antibody, arbitrarily named Fab7816, able to react both with coronary and carotid atherosclerotic tissue samples. We also demonstrated by confocal microscopy that this monoclonal antibody recognized human transgelin type 1, a cytoskeleton protein involved in atherogenesis, and that it co-localized with fibrocyte-like cells transgelin+, CD68+, CD45+ in human sections of coronary and carotid plaques. In vitro fibrocytes obtained by differentiating CD14+ cells isolated from peripheral blood mononuclear cells also interacted with Fab7816, thus supporting the hypothesis of a specific recognition of fibrocytes into the atherosclerotic lesions. Interestingly, the same antibody, cross-reacted with the outer membrane proteins of *Proteus mirabilis* and *Klebsiella pneumoniae* (and possibly with homologous proteins of other *enterobacteriaceae* present in the microbiota). From all the other three libraries, we were able to clone, by immunoaffinity selection, human monoclonal antibodies cross-reacting with bacterial outer membrane proteins and with transgelin. These findings demonstrated that in human atherosclerotic plaques a local cross-reactive immune response takes place.

## Introduction

Atherosclerosis is a slowly progressing disease with an inflammatory component in which the adaptive immunity is directly involved since its early stages down to the progression and acute degeneration of advanced lesions [1]–[2]. Targeted biological treatment for coronary and carotid atherosclerotic diseases is still limited and this is mainly due to their complex pathogenesis being still far from being fully understood. B cells have already been described both in animal models and in human atherosclerotic lesions, where they can display an organization resembling tertiary lymphoid organs [3], [4], [5] which sustain a chronic proinflammatory environment [6]. We have recently documented an oligoclonal distribution of B cells in atherosclerotic plaques with the molecular evidence of an antigen-driven B cell maturation within human coronary lesions [7]. Interestingly, in patients with acute coronary syndrome (ACS), an oligoclonal population of T cells in unstable coronary plaques was also described, further suggesting the persistence of local targets of the immune response [8]. Previous demonstration in the coronary plaque of a local B-cell and T-cell response suggested that exogenous agents including bacteria and viruses may play a causal role in the local inflammation [9]. Incompletely understood microbes-host interactions have been associated with the initiation, perpetuation and re-exacerbation of atherosclerotic lesions eventually leading to thrombus formation and acute coronary syndromes or stroke [10], [11], [12], [13], [14], [15]. Recent studies showed that atheromas collect bacteria from the circulation and microbial molecular signatures have been detected in progressively higher frequency in advanced lesions [16], [17]. Nevertheless, even if many observations suggest an involvement of several microbes in the pathogenesis of atherosclerosis, definitive exogenous players have not been clearly identified yet [11], [12], [18], [19]. In parallel, endogenous triggers (such as oxLDL) may play a role in the pathogenesis of coronary plaques, in analogy with the chronic inflammatory processes observed in autoimmune diseases [9], [20], [21], [22].

**Figure 1:**
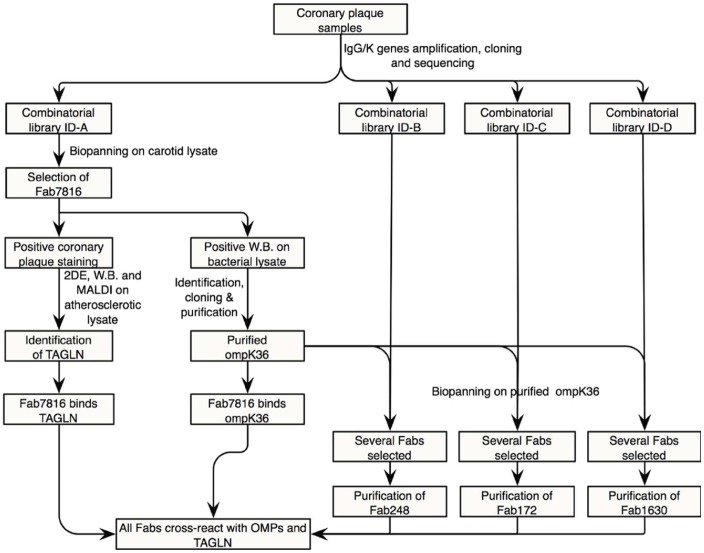
Study flow-chart. TAGLN  =  transgelin; OMPs  =  outer membrane proteins.

We now demonstrate, by molecular cloning in a phage display library of the IgG1/k repertoire present in coronary plaques and subsequent generation of human monoclonal antibodies, that B cells in the plaques of four different ACS patients produce antibody clones cross-reacting with the OMPs of gram- bacteria (*K. pneumoniae* and *P mirabilis*) and with transgelin (TAGLN), a cytoskeleton protein present in adult human smooth muscle cells (SMC) and other cell types (Figure 1) [23]. We also demonstrate that these antibodies recognized TAGLN expressed in cells with the phenotypic features of fibrocytes, a monocyte-derived-cell population present in coronary and carotid plaque sections. These cells are plastic monocyte-derived cells that migrate and differentiate under chronic inflammatory stimuli as previously observed in several autoimmune conditions such as scleroderma, autoimmune thyroiditis and rheumatoid arthritis [24]. The identification of local and exogenous antigenic stimuli recognized by the local adaptive immune response in human lesions explains the reason for the active recruitment of B cells in human lesions and has the potential of opening new vistas in the understanding of their role in the pathogenesis of acute coronary disease and to plan novel strategy in order to modulate the inflammatory component of the atherosclerotic process.

## Results

### Combinatorial Libraries Construction and Selection of Fab7816 from Biopanning with Library ID-A on Atherosclerotic Carotid Lysates

Four combinatorial antibody Fab fragment phage-display libraries were generated from four coronary plaques (ID-A, ID-B, ID-C and ID-D). Clinical characteristics of the four patients from which the plaques were obtained were recorded and shown in table S1. In particular, each patient was showing an heterogeneous combination of known risk factors for cardiovascular events such as family history, hypertension, smoking and high cholesterol levels and the analyzed plaques were causing an average 67.75% coronary lumen reduction. Three patients entered the coronary unit with the clinical manifestation of an infarction and another one with the diagnosis of unstable angina.

After generation of combinatorial phage-display libraries, sequencing was performed on a sample of clones and results of heavy and light chains analyses are shown in table S2. Results from the preliminary screening performed on all the libraries were compatible with the oligoclonality previously observed in coronary samples and with a limited number of clones with different junctions (meaning distinct B-cell receptors) [7]. Size of all libraries guaranteed all possible heavy and light chain combinations present in each phage display library. Apparently no preferential V gene usage was observed and all heavy and light chain genes were highly mutated, as determined by comparison with germline sequences (table S2). Library ID-A showed the highest number of clones with different junctions both in the light and in the heavy chains at sequence screening and was thus chosen for immunoaffinity selection against atherosclerotic carotid plaque lysates.

**Figure 2:**
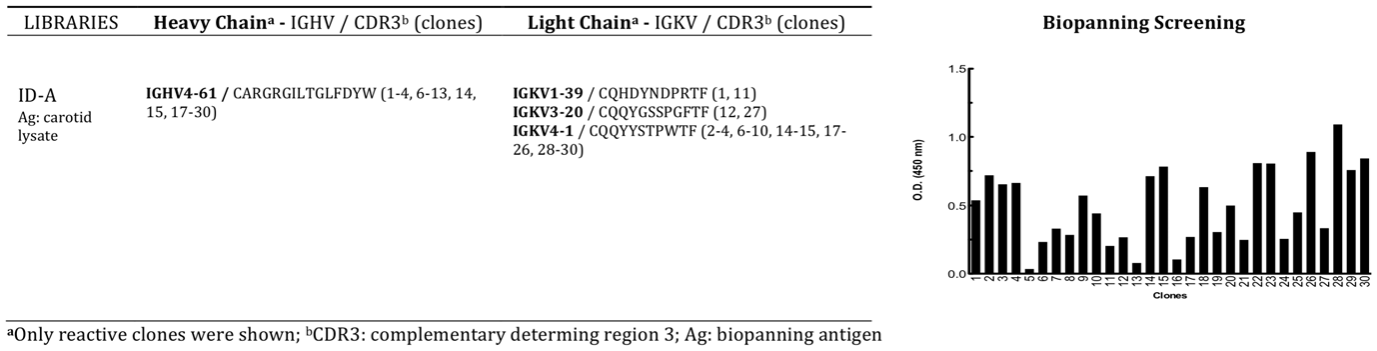
Biopanning selection with combinatorial IgG/k library ID-A. Library ID-A was selected by immunoaffinity on atherosclerotic plaque lysate. Results of screening ELISA assays of 30 clones after four rounds of biopanning selection is shown. Sequence analysis of the positively selected clones (O.D.450 nm >0.25 above background) is shown next to the ELISA screening.

After four rounds of biopanning, the procedure was stopped, the eluted phages recovered and the phage-displayed Fabs were solubilised in order to confirm atherosclerotic carotid plaque lysates binding. Thirty individually selected Fab preparations were screened in ELISA on carotid lysates and each phagemidic vector was sequenced (Figure 2). The majority of clones were bearing an identical combination of heavy and light chain genes (with the IGHV4-61 and IGKV4-1 minigenes respectively) coding for a Fab that we named Fab7816 (Figure 2). Fab 7816 was purified, then used for identification of the antigens recognized in the carotid lysate.

### Fab7816 Recognizes TAGLN as Putative Natural Self-antigen in Atherosclerotic Lesions

**Figure 3:**
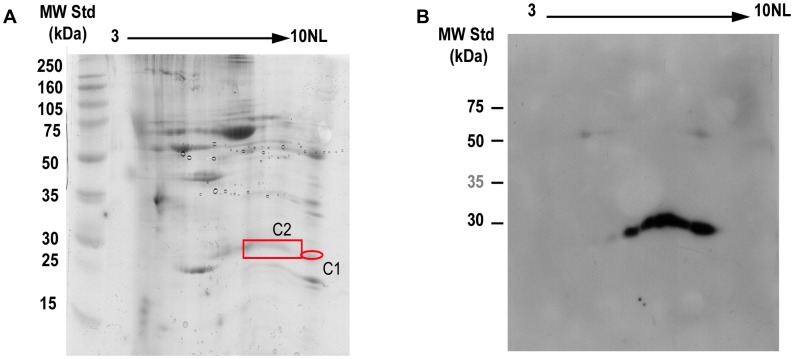
Identification of putative natural self antigen. A) Bidimensional electrophoresis gel stained with colloidal Coomassie Brilliant Blue. Sixty five **µ**g of purified carotid atherosclerotic plaque proteins were loaded on strip pH 3-10NL, 7 cm. The 2nd dimension was carried out using 12.5% SDS-PAGE. B) 2D electrophoresis Western Blotting. Similarly, 70 **µ**g of proteins were loaded on strip pH 3-10NL, 7 cm. The 2nd dimension was carried out using 12.5% SDS-PAGE. After transfer, proteins were probed with Fab7816-FLAG (10** µ**g/mL). Protein spots of interest (red box) were excised from the gel, digested with trypsin and analysed by MALDI-ToF mass spectrometry. In both spots human TAGLN was identified with almost complete sequence coverage.

The protein present in human atherosclerotic carotid preparations that was recognized by Fab7816 was identified by Bidimensional electrophoresis (2DE) of carotid plaque lysates (Figure 3) and by mass spectrometry analysis on proteins sampled from two distinct spots in the 2D gel. These experiment unequivocally identified TAGLN as the putative antigen recognized by Fab7816 with almost complete protein coverage of TAGLN in mass spectrometry analysis. 2D gel showed that possibly more than one TAGLN isoforms, with different Ip, were recognized by Fab7816. In fact in both spots of the 2D gel, TAGLN was recognized by Fab7816 (Figure 3B).

### Fab7816-FLAG Stains a Cell Subset with the Phenotypic Features of Fibrocytes in Atherosclerotic Coronary and Carotid Tissues Samples and Recognize Human Fibrocytes in vitro

**Figure 4:**
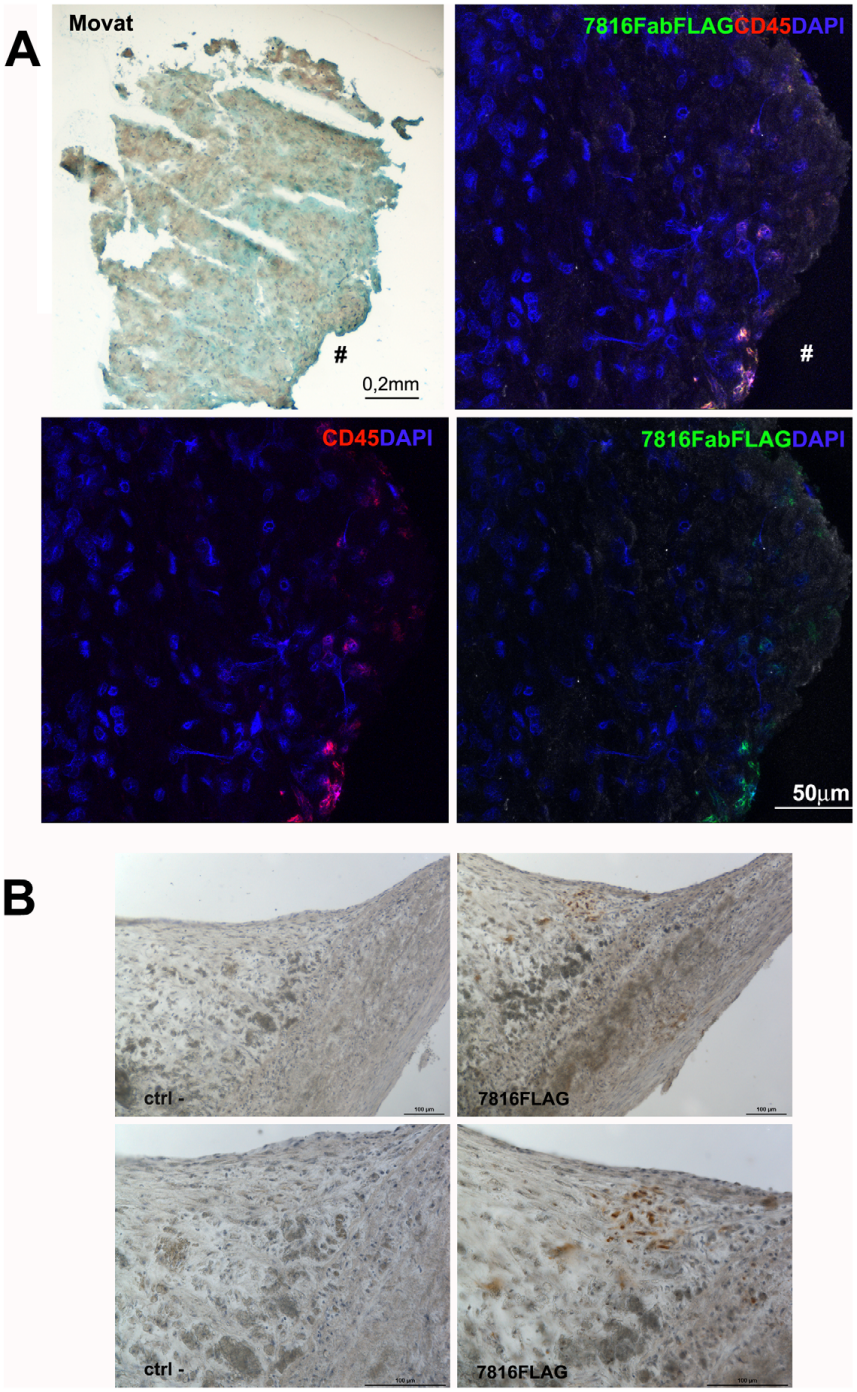
Immunofluorescence on human coronary plaqes and Immunohistochemistry on human carotid sections with Fab7816-FLAG. a) Representative section stained by Movat’s pentchrome (left panel) showed the morphology of a portion of the coronary plaque tissue (plaque ID-A) displaying slightly damaged media rich in smooth muscle cells. Confocal microscopy (right panels) showed the presence of CD45^+^ cells (red) labelled by Fab7816-FLAG revealed by MAb M2 anti-FLAG-FITC (green) in the coronary plaque tissue region indicated by symbol (#). DAPI stained the nuclei (blue). b) Immunoperoxidase on carotid plaque samples demonstrated the presence of several cells reacting with Fab7816-FLAG in an area close to the lumen (left panels) as revealed by MAb M2 anti-FLAG-HRP developed with DAB (brown). The signal is absent in a serial section where the Fab7816-FLAG is omitted (ctrl-, right panels). Magnified images in the bottom panels evidence the spindle shape of Fab7816-FLAG+ cells and their localization in between a clusters of altered cells, possibly foam cell. Haematoxylin (blue) stains nuclei. Scale bars indicate the magnification.

**Figure 5:**
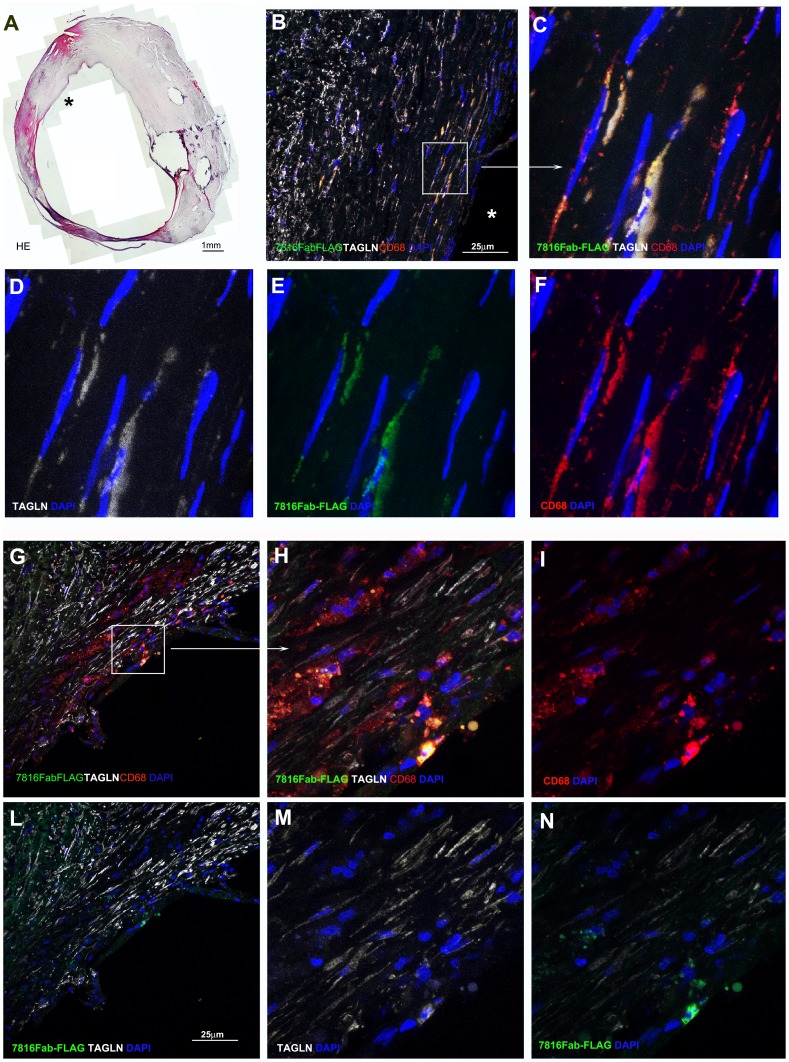
Confocal microscopy on human atherosclerotic carotid sections. Multiple staining of two carotid plaques are displayed to characterize the Fab7816-FLAG+ cells type. First plaque A-F panels, 2^nd^ plaque G-I to N. The reconstruction of a section from 1^st^ plaque stained with haematoxylin and eosin (HE) obtained by multiple images grabbing tool of Lucia-G software is in A; asterisk indicates the lumen in correspondence of Fab7816-FLAG+ cells, on the shoulder of the atheromasic lesion demonstrated in B by confocal lmicroscopy. Confocal microscopy images in B,C and G,H demonstrated by Fab7816-FLAG (green), goat-anti-human TAGLN (white), mouse-anti-human CD68 (red) the presence of triple positive cells in the intima, closely to the lumen. Single or double staining are shown in D-F and I-N. Squares and arrows indicated the enlarged areas. DAPI stains the nuclei (blue). Scale bars indicate the magnification.

**Figure 6:**
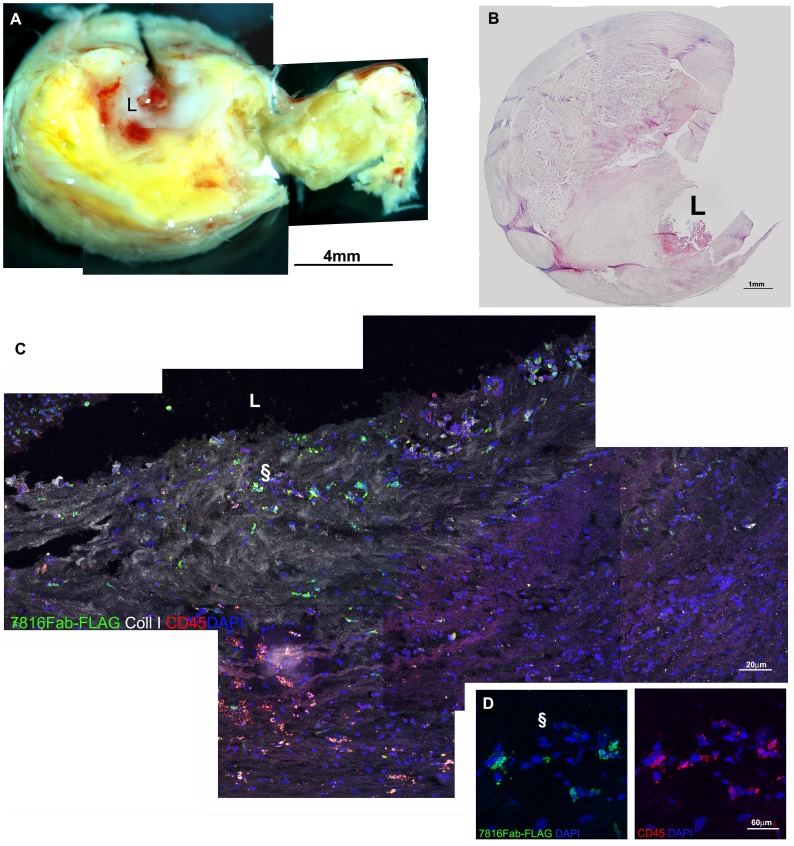
Confocal microscopy on human atherosclerotic carotid sections. The macroscopic aspect of a carotid plaque is reconstructed in A by stereomicroscope and displays a lipidic core and of luminal thrombus. Features are confirmed in B by histology (haematoxylin and eosin). Confocal microscopy in C shows a reconstruction of part of the area close to the lumen (L) in a section stained with Fab7816-FLAG (green), mouse-anti-human Collagen type I (white) and mouse anti-human CD45 (red), revealed by opportune secondary antibodies. In D is an enlargement of the area indicated by § symbol in C with several Fab7816-FLAG+/CD45+ cells in the neointima. indicate a regions magnified. Scale bars indicate the magnification.

**Figure 7:**
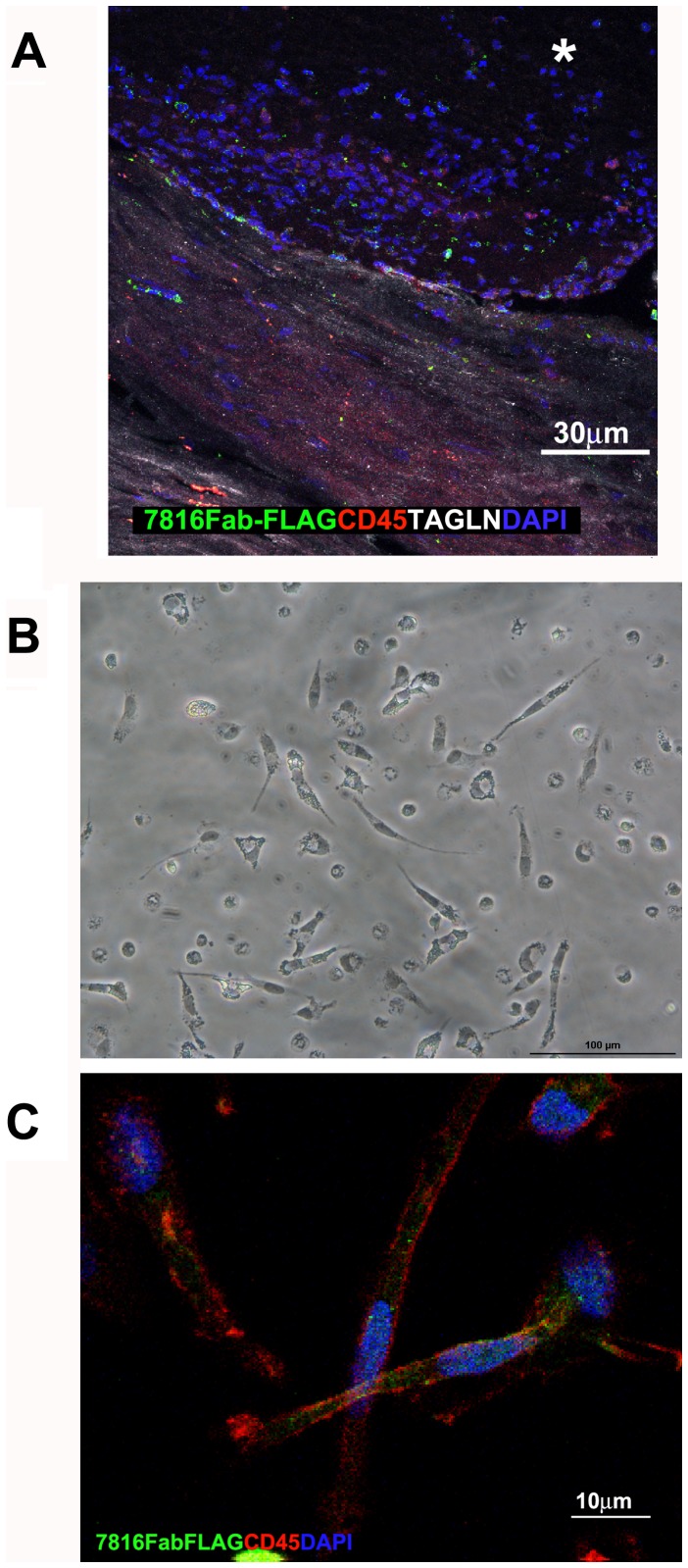
Fibrocytes morphology and immunoreactivity with 7816Fab FLAG. A) Confocal microscopy staining showing presence of 7816Fab FLAG +/CD45+/TAGLN+ cells in human carotid plaque lesion. B,C) Confocal microscopy image of non-confluent CD14+ fibrocytes grown on glass for 4 days in the absence of serum. Spindle shaped cells are stained with 7816Fab FLAG +/CD45+(green and red, respectively). DAPI stains the nuclei (blue). Scale bars indicate the magnification.

Confocal microscopy analysis on human coronary plaque sections showed specific interaction of Fab7816-FLAG with cellular antigens present in the tissue from its originary coronary plaque (plaque ID-A) (Figure 4a), as well as with cells localized under the fibrous cap into a carotid plaques (Figure 4b). Anti Hepatitis C virus human monoclonal E8Fab-FLAG [25], used as a negative control, failed to show any specific signal (Figure S1). A subset of TAGLN+/CD45+ cells was recognized by Fab7816-FLAG in human coronary samples (Figure 4a). To further characterize the binding capability of Fab7816-FLAG to atherosclerotic tissue antigens, carotid plaque samples were used (Figure 4b, 5,6 and 7). Specific interaction of Fab7816-FLAG but not of E8Fab-FLAG was observed. Fab7816-FLAG labelled carotid plaque antigens localized in the intima (Figure 4,5,6) in 19 patients out of 31 (table S3), and more rarely in the sub-adventitia (not shown). In our limited series of patients no correlation between the presence of an mmunoreactivity vs. Fab7816-FLAG and the histological features of carotid plaque was found (table S3). Double and multiple staining were performed. Double staining with Fab7816-FLAG and anti- TAGLN antibodies (either monoclonal or polyclonal) demonstrated the existence of Fab7816-FLAG+/TAGLN+, (figures S1, S2, S3 and negative controls figure S4) and of Fab7816-FLAG+/CD68+ cells into the carotid plaque (figure S2). By multiple staining the Fab7816-FLAG+ appeared TAGLN+/CD68+ (Figure 5 and negative samples figure S4), and Coll type I+/CD45+ (Figure 6). Moreover triple labelling showed that Fab7816-FLAG+ cells are TAGLN+/CD45+, thus demonstrating that Fab7816-FLAG recognized in the atherosclerotic lesions a subset of cells of monocytoid origin and with a fibrocyte phenotype. In vitro culture of fibrocytes differentiated from CD14+ circulating monocytes from healthy donors (figure 7a) and displaying the main phenotypic markers (figure S5A,B) demonstrated the presence Fab7816-FLAG specific binding in CD45+ cells (Figure 7b). further confirming the specificity of Fab7816-FLAG for fibrocytes.

### Fab7816 Cross-reacts with OmpK36 and OmpF

**Figure 8:**
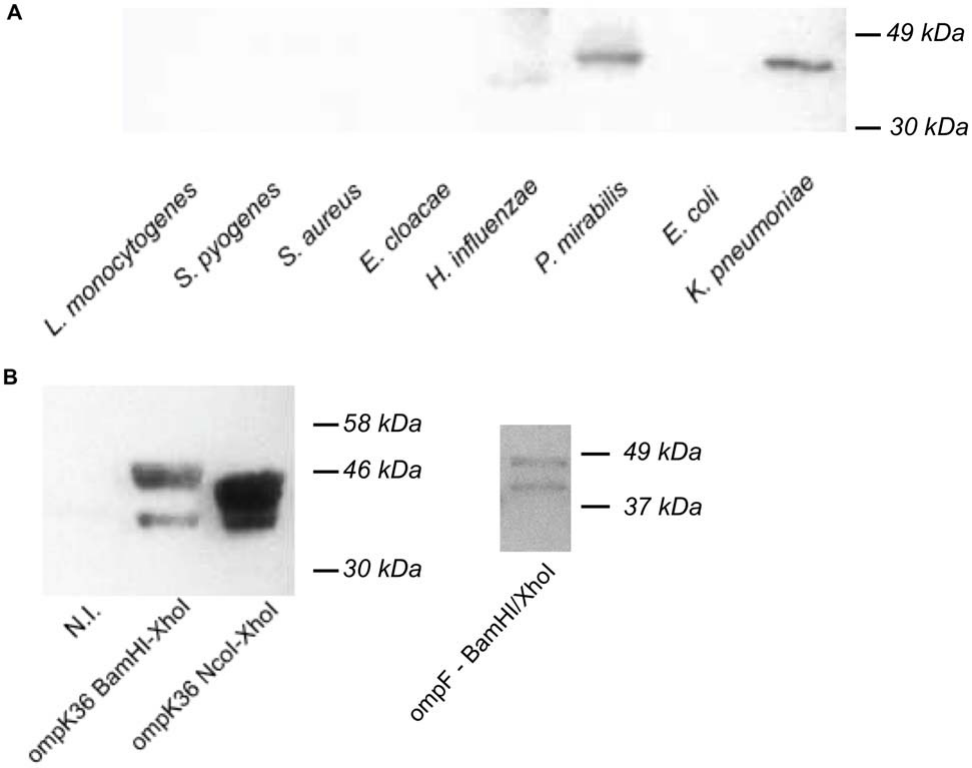
Western Blotting of bacterial lysates and on bacterial OMPs with Fab 7816. A) Western blotting of bacterial lysates with Fab7816. 1** µ**g of each bacterial lysate was loaded in each lane. Fab 7816 clearly reacted with *Proteus mirabilis* and *Klebsiella pneumoniae* lysates. B) Western blotting on induced/non induced BL21(DE3) bacterial cells transformed with pET28b vector expressing OmpK36. Two pET vectors were constructed: the NcoI/XhoI wild type OmpK36 vector or the HIS-tagged Ompk36 BamHI/XhoI vector. In both cases Fab 7816 recognized only IPTG induced bacteria.

Purified Fab7816 was also tested for its capability to bind and to identify potentially exogenous antigens by screening bacterial lysates in western blot (WB). Fab7816 recognized a protein band of about 35 kD in *Proteus mirabilis* and *Klebsiella pneumoniae* lysates. (Figure 8) Among the proteins of *Klebsiella pneumoniae* with this molecular weight, we cloned and purified the major outer membrane protein (OmpK36). OmpK36 was expressed in *E. coli BL21(DE3)* strain (Figure 8A) and Fab7816 staining of induced *E. coli BL21(DE3)* demonstrated specific binding of Fab7816 to OmpK36. Binding experiments on cloned and purified outer membrane protein F (OmpF) of *P.mirabilis* confirmed that Fab 7816 is able to recognize also the homologous target in *P.mirabilis* lysate (OmpF) (Figure 8b).

### Antibodies Similar to Fab7816, Recognizing TAGLN and Outer Membrane Proteins (OMPs), are Present in the Locally Produced Antibody Repertoire in Other Three Coronary Plaques from Distinct Patients

**Figure 9:**
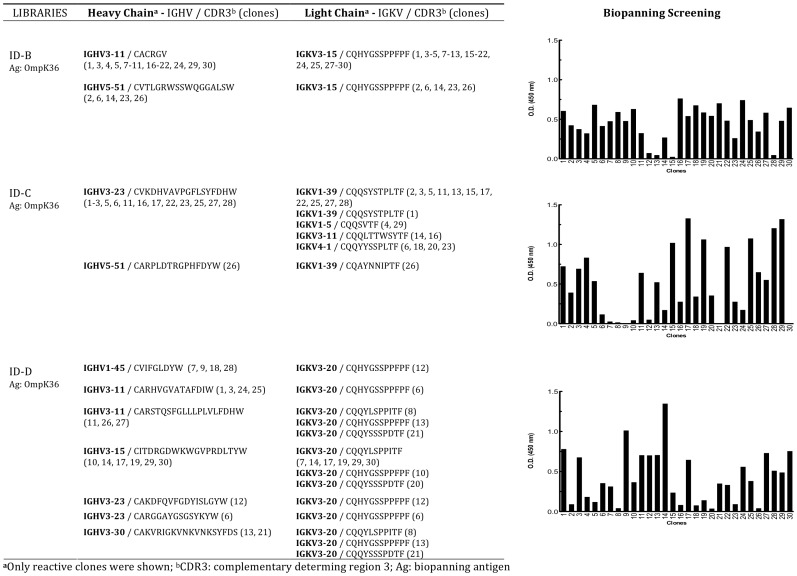
Biopanning selection with combinatorial IgG/k libraries ID-B, ID-C and ID-D. Each library was independently selected by immunoaffinity on purified OmpK36. Results of screening ELISA assays of 30 clones after four rounds of biopanning selection is shown. Sequence analysis of the positively selected clones (O.D.450 nm >0.25 above background) is shown next to each ELISA screening.

To prove that the local production of antibody clones crossreacting with TAGLN and OMPs in atherosclerotic plaque ID-A was not incidental, nor a single lesion-related finding, the biopanning selection was performed with all three additional libraries from three distinct patients on purified OmpK36 (figure 1 and table S2) since Fab7816 reacted poorly on commercial purified TAGLN in ELISA (Figure S6), OmpK36 was preferred for immunoaffinity selection with the other three libraries. After four independent selection rounds with libraries ID-B, ID-C or ID-D on purified OmpK36, biopanning was stopped and 30 single clones for each library where screened in ELISA on purified OmpK36 and sequenced (Figure 9). Several distinct Fabs, bearing different combinations of heavy and light chains, but all able to specifically bind *K.pneumoniae* OmpK36, were cloned from each library. For each plaque either the most represented among the selected clones or the best reacting Fab at screening was produced and purified. The obtained representative Fabs were named Fab248 (from plaque ID-B), Fab172 (from plaque ID-C) and Fab1630 (from plaque ID-D).

### All Representative Fabs Recognised in Purified Human TAGLN and Bacterial OMPs

**Figure 10:**
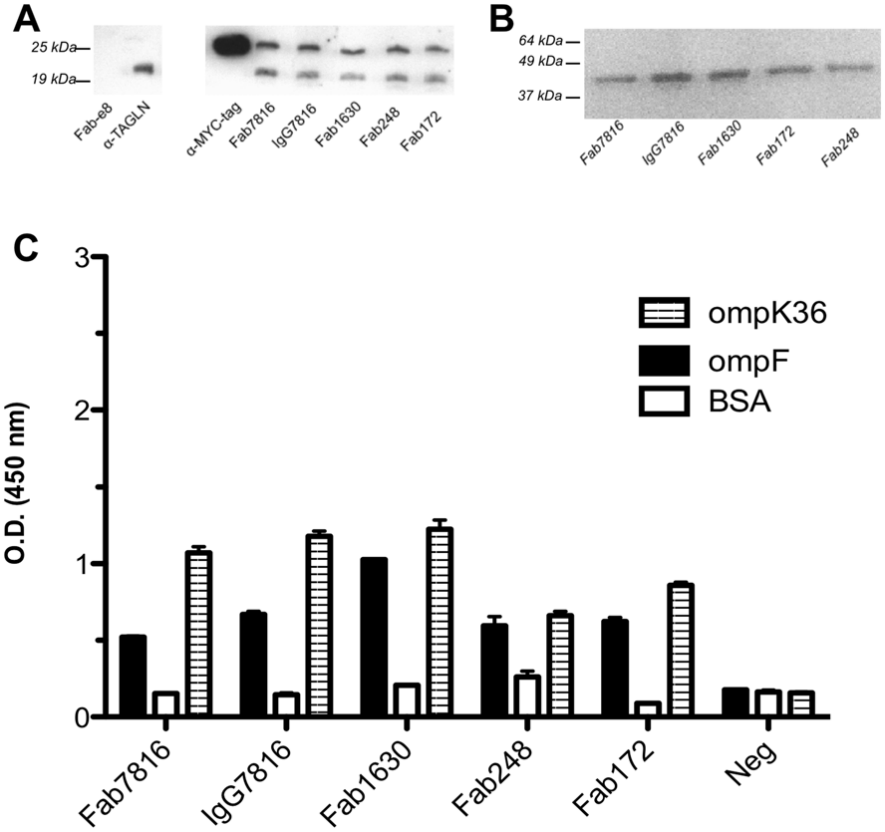
Cross reactivity of representative Fabs from all patients with TAGLN and OMPs. A) WB of purified human TAGLN (400 ng) with all representative Fabs (10** µ**g/mL). Unrelated human e8Fab-FLAGwas used as negative control. Anti-MYC-tag (C-terminal tag) and commercial anti-TAGLN were used as positive controls. While the commercial anti-TAGLN recognize selectively only one form of TAGLN, cloned human Fabs recognized both forms. B) WB of OmpK36 (500 ng) with all representative Fabs (10 ng/mL). C) ELISA with all representative Fabs on bacterial OMPs. Reactivity against bovine serum albumin (BSA) used as blocking antigen, is also shown.

All representative Fabs displayed a strong binding and cross-reacting capability against both OMPs and human TAGLN (Figure 10). In particular, ELISA and WB experiments, conducted using purified commercial human TAGLN preparations showed that all Fabs bind to TAGLN in WB (FIG. 10a) but not in ELISA (Figure S6) as initially observed with Fab7816 (figure S6). Since electrophoresis was conducted under denaturing conditions, it is very likely that Fabs recognized a linear epitope of the TAGLN protein that is poorly exposed in these ELISA conditions, whilst fully accessible in WB under denaturing conditions (see also Figure 3b). Reactivity was also verified with Fab7816 expressed and purified as full IgG (IgG7816) (protocol S1) (figure 10).

All representative Fabs confirmed their binding both to *Proteus mirabilis* and *Klebsiella pneumoniae* purified OMPs in WB (only WB on OmpK36 is shown in Figure 10b) and in ELISA (figure 10c). Reactivity was also verified with Fab7816 expressed and purified as full IgG (IgG7816) (figure 10b and 10c). To confirm that binding followed an antigen-driven affinity maturation and to exclude the superantigenic nature [26] of OMPs binding by our Fabs, the germline (GL) reverted Fab7816 (protocol S2) was tested against the purified bacterial antigens. Purified Fab7816GL did not recognize OMPs (data not shown).

### Commercial Monoclonal Antibodies Against Human TAGLN Cross-react with *Klebsiella pneumoniae* OmpK36 and *P.mirabilis* OmpF

**Figure 11:**
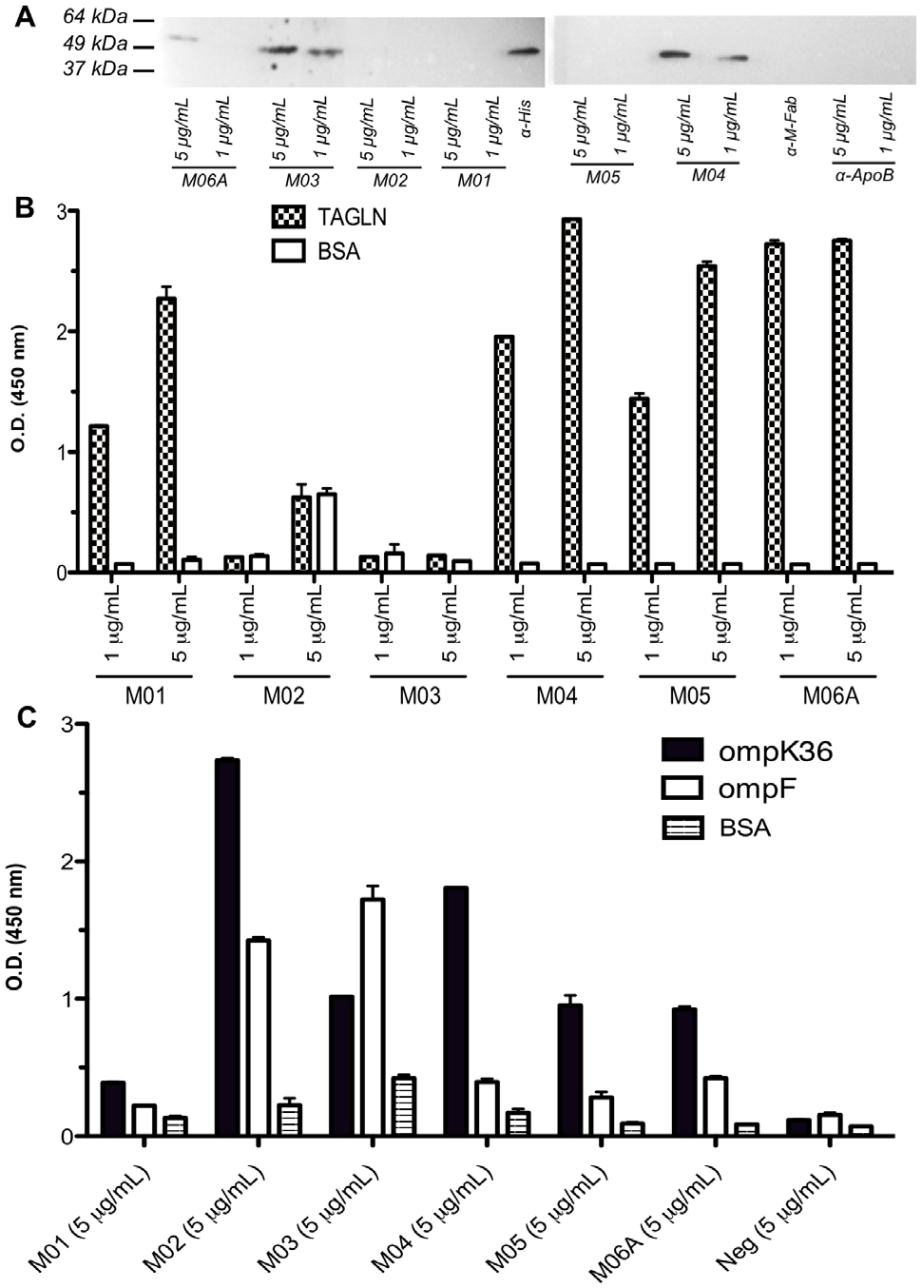
Cross reactivity of commercial available monoclonal antibodies with TAGLN and OMPs. A) WB of OmpK36 with five commercial monoclonal mouse anti-human TAGLN antibodies (used at 1 or 5 **µ**g/ml). Three commercial mouse monoclonal antibodies were used as negative controls. Anti-6xHIS antibody (Roche) was used as positive control B) ELISA with all representative and five commercial monoclonal mouse anti-human TAGLN antibodies on purified human TAGLN or C) bacterial OMPs. Reactivity against bovine serum albumin (BSA) used as blocking antigen, is also shown.

Five different commercial monoclonal mouse anti-human TAGLN IgG antibodies (Abnova, H00006876-M01, M02, M03, M04, M05) and a monoclonal mouse anti-human TAGLN IgM antibody (Abnova, H00006876-M06A) were tested for their potential cross-reactivity against human TAGLN in WB (Figure 11a) and in ELISA (Figure 11b) and against bacterial OMPs (both OmpK36 and OmpF) in ELISA (Figure 11c). While all commercial monoclonal antibodies recognized TAGLN and purified OmpK36 and OmpF in ELISA (although apparently with distinct efficiencies), three of them, M03, M04 and M06, recognized TAGLN also in WB further con confirming the existence of a cross-mimicry between human TAGLN and the bacterial OMPs.

## Discussion

In a previous work [7] we demonstrated that in human coronary atherosclerotic plaques an antigen-driven B-cell evolution takes place, indicating the local presence of an antigen that stimulates the immune system. We now show that in human coronary atherosclerotic plaques B-cells are indeed engaged by local antigens and produce antibodies clones able to cross-react both with the fibrocyte cytoskeleton protein transgelin and with antigenic determinants of the outer membrane proteins of gram-negative pathogens belonging to the *enterobacteriacee* family. This original observation unequivocally demonstrate the reason for the recruitment of a population of B cells in human plaques. Although our observation lacks the formal demonstration of a direct pathogenetic or a protective role of this process, the molecular characterization of immunological targets is crucial for the understanding of a complex disease in witch the adaptive immune response plays an important role in all its phases of development [2]. Moreover, several previous observations could be re-analyzed in light of this observation. Indeed this finding could also explain why, despite some retrospective and cross-sectional sero-epidemiological studies found a strong association between distinct bacterial infections and coronary artery disease [12], [15], [17], [18], [27], [28], [29], specific pathogens where rarely detected in the arterial vessel walls. Indeed, this work opens the possibility of considering in the aetiopathology of the atherosclerotic disease a “hit-and-run” mechanism, in which the antigenic stimulations due to a self antigen or to the intermittent circulation of bacterial particles may persist, even in absence of a local replication [12], [30], [31]. It can be speculated that in atherosclerotic plaques both acute infections or even the mere spreading of gram- bacterial antigens, even from distant sites, may trigger rapid inflammatory changes and specific T- and B-cell responses within days or weeks [11], [14], [15] in spite of the local absence of the microbe itself, thus influencing the status of the local disease. In this model, a role in the progression of the atherosclerotic disease can also be played by low density lipoprotein (LDL) microparticles or outer membrane vesicles that can vehicle bacterial OMPs from the circulation to the site of atherosclerotic plaque growth [32], [33]. In this view our work can also explain why periodontitis, characterized by transient bacteraemia originated from the oral lesion, seems to be a risk factor for cardiovascular disease [34]; or why infections of the upper respiratory and urinary tracts due to gram-negative bacteria belonging to the *enterobacteriaceae* family represent, especially in the elderly and for diabetic patients, a risk factor for AMI or stroke [2], [11], [14], [15]. Antigenic mimicry between microbial and self structures has already been described [16], [22], [31] but our work provides for the first time a molecular dissection and characterization of this still elusive phenomenon, showing that within the plaques self- (TAGLN) and exogenous- (OMPs) antigens are recognized by highly mutated class-switched antibodies.

The nature of the two antigens recognized by the antibodies locally produced in the atherosclerotic plaque requires some additional considerations. OmpK36 (and possibly other OMPs) was demonstrated to activate immune cells via Toll-like receptors as well as antigen-presenting cells including B cells, gaining access to the major histocompatibility complex class I pathway and triggering the initiation of cytotoxic responses even in the absence of CD4^+^ T-cells help [35], [36]. On the other hand TAGLN has already been demonstrated to play a role in atherosclerosis. It was shown that in ApoE-deficient mice the ablation of TAGLN, increases the plaque area by enhancing the SMC phenotypic shift from contractile to synthetic/proliferative [37], [38]. Furthermore, disruption of TAGLN promotes arterial inflammation in mice through reactive oxygen species (ROS)-mediated NF-κB pathways [37], [39], increases atherosclerotic lesion area and favours SMC transdifferentiation into foam cell [37]. However, in human carotid plaques, most of the TAGLN+ cells were not recognized by cross-reactive antibodies produced in coronary plaques, as Fab7816. This may suggest that epitope accessibility in these cells, or specific antigenic modifications or intracellular processing occur in this cell population that are necessary for auto-antibody binding in human lesions. This cell population has the molecular feature of a subpopulation of monocyte-derived cells named fibrocytes. This cells have been previously identified in the fibrous caps of human atheromas and in several animal models of peripheral vascular disease. Although these cell type share molecular markers of either macrophages or fibroblasts, they remain relatively undifferentiated and express CD45 (also observed in our plaque samples) [40]. As suggested by animal models, fibrocytes might contribute to the pathogenesis of ischemic cardiomyopathy and might have a protective or reparative effect in the setting of uncontrolled or persistent in vivo TH1 cell cytokine exposure, whereas in response to persistent TH2 cell cytokine exposure (as during B-cell stimulation) they become profibrotic [24]. Whether locally produced cross-reacting IgGs play a specific role in any of these events, is still to be elucidated. Noteworthy, only a number of the atherosclerotic carotid samples were Fab7816+, thus suggesting that this mechanism might specifically identify a distinct plaque phenotype. Further studies are required to establish a relationship between Fab7816 staining and plaque features or clinical characteristics, in order to stratify patients.

However, the demonstration that, in atherosclerotic plaques of ACS patients, antibodies that cross-react with a bacterial antigen and with a self protein involved in atherogenesis together with the fact that these antibodies seem to recognize TAGLN in lesions from a number of unrelated patients opens new perspectives in understanding the role played by the adaptive immune system in the pathogenesis of the atherosclerotic inflammatory processes, providing a scientific ground to several clinical and pathological features of the disease. In theory, the continuous and transient exposure to exogenous cross-reacting molecules during asymptomatic bacteraemia events can guarantee the surviving stimuli to the self-reactive immune cells within the lesions [6]. Moreover, self antigens produced by dying cells or neo-epitopes generated by LDL oxidation (oxLDL) together with the development of autoantibodies against oxLDL have been recently suggested as pro-atherogenic events [2], [41]. A similar mechanism involving cross-reactivity between commensal microbiota and self structures was also recently demonstrated to play pathogenetic role in mice models of autoimmune demyelination [22].

Unfortunately, formal demonstration of the aetiopatological role of the molecular mimicry we describe may not be reliably reproduced in animals, due to the difficulty of reproducing human autoimmune diseases in experimental models. Current animal models of atherosclerosis do not fully mimic human disease (with particular regard to coronary atherosclerosis and AMI) and the immune response eventually generated by vaccination would not necessarily reflect the role of the local immune response present within the lesions.

As a conclusion, the availability of these data could be central for planning new strategies to correctly address the role of immune response in coronary atherosclerosis. The identification of exogenous and endogenous triggers may help to identify new therapies, such as inducing local B-cell local apoptosis or depletion by targeting B-cell receptor signalling with the identified antigens or synthetic mimotopes; or driving B or T-cell function towards a regulatory phenotype to inhibit the inflammatory cascade [1]. The immunization strategies with specific exogenous antigens may prevent bacterial immunogens from reaching atherosclerotic lesions. Moreover, a more precise identification of the nature of the epitopes involved in this complex process has the potential of allowing the design of novel diagnostic assays that may be able to predict the disease status in atherosclerotic patients and to help to define the risk for disease progression.

## Methods

### Ethics Statement

The San Raffaele ethics committee approved the study and all patients gave their written informed consent to participate.

### Samples Collection and Amplification of Heavy and Light Chain Genes

Four coronary atherosclerotic plaques (ID-A, ID-B, ID-C, ID-D) were collected and stored in liquid nitrogen from four male patients with ACS (Table S1), undergoing coronary atherectomy. None of the patients had autoimmune or systemic diseases or diabetes, nor had they undergone recent surgery or suffered for from recent infectious diseases. Plaques were homogenized and total mRNA was extracted using RNeasy Micro kit (Qiagen) according to the manufacturer’s instructions. Amplification of heavy and light chain genes was performed as previously described [7].

### Molecular Cloning and Combinatorial Antibody Fab Fragment Phage-display Libraries

The PCR products of heavy and light chains amplified from human coronary plaques were cloned into a phagemidic vector (pRB32) that allowed the generation of combinatorial antibody Fab fragment phage-display libraries [7], [42], [43].

### Lysates Preparation from Atherosclerotic Carotid Samples and from Bacterial Pathogens

Human atherosclerotic carotid artery biopsies from patients submitted to endoarterectomy were collected in lysis buffer (RIPA buffer), homogenized with Tissue Ruptor (Qiagen) and centrifuged at 13,000 rpm for 30 min at 15°C. Supernatant protein content was evaluated by BCA (Pierce). To prepare bacterial lysates, *Staphylococcus aureus, Proteus mirabilis, Klebsiella pneumoniae, Enterococcus cloacae, Streptococcus pyogenes, Neisseriae gonhorreae, Listeria monocytogenes*, *Haemophilus influenzae* were inoculated in 10 mL of super broth or seeded on blood agar, if required, and grown at 37°C overnight. Cultures were harvested by centrifugation and resuspended in RIPA buffer containing a protease inhibitor (phenylmethylsulfonyl fluoride (PMSF). After a sonication step the lysate was cleared by centrifugation and stored at −20°C until usage.

### Immunoaffinity Selection by Biopanning on Atherosclerotic Carotid Lysate

The night before biopanning, the ELISA plate was coated O/N at 4°C with the carotid lysate (100 ng/well) solution in a coating buffer (PBS, pH = 7.4). After blocking with 3% bovine serum albumine (BSA) in phosphate buffer solution (PBS), for 1 h, 37°C, 70 µL of phage suspension, (about 10^11^ PFU), were added to each well and incubated for at least 2 hrs at 37°C. Selected phages were recovered and used to infect a fresh *E. coli XL-1Blue* culture. Biopanning was then conducted as previously described [25] for four rounds, allowing enrichment of the selected population.

### Production and Purification of Fabs

*E. coli XL-1Blue* was used for the expression of soluble Fabs as previously described [25].

### Production and Purification of *Klebsiella pneumoniae* OmpK36 and *Proteus mirabilis* OmpF

OmpK36, and OmpF were amplified from *Klebsiella pneumoniae* and *Proteus mirabilis* lysates respectively and cloned into a pET-28b expression vector (Novagen) by using the following primer pairs containing two distinct 5′ restriction endonuclease sequences: OmpK36NcoIFW: GCGCTAGTATGccatgggcATGAAAGTTAAAGTACTGT and OmpK36XhoIRW: GCGCTTCCTCGATACCTCGAGCTAGAACTGGTAAA; OmpK36BamHIFW: GCGCTAGTCTGggatccgATGAAAGTTAAAGTACTGTC and OmpK36XhoIRW: TTCCTCGATACCTCGAGCTAGAACTGGTAAACCAG; OmpF-Pr-BamHI TGATCGATCGAAATGggatccgATGATGAAGCGCAATAT and OmpF-Pr-XhoI GTA CCT AAT CCAGCAACGctcgagGAACTGATAAGT. For the amplifications the following thermal profile was used: 95°C for 5 min; 95°C for 30 sec., 55°C for 30 sec. and 72°C for 1.5 min. for 30 cycles; 72°C for 10 min. The amplification products and the pET28b vectors were double digested for 4 hours with the following couples of restriction endonucleases: NcoI/XhoI (New England Biolabs) or BamHI/XhoI (New England Biolabs) for OmpK36 and only BamHI/XhoI for OmpF, loaded onto a 1% agarose gel and purified by using Qiagen Gel Extraction kit. The corresponding digested amplicons were overnight ligated by using 10 units of T4 ligase (Roche). After transformation of bacterial cells (E. coli XL1-Blue) and plating, individual colonies were inoculated on Luria Broth (LB) and grown overnight. After plasmid purification (Qiagen Miniprep kit) the inserts were sequenced with the following primers: T7FWpET: TAATACGACTCACTATAGGG and T7termpET: GCTAGTTATTGCTCAGCGG. OMP Expression was performed following manufacturer instructions (pET System Manual - Novagen), using a final IPTG concentration of 1 mM to induce the expression of OMPs. After induction, cultures were chilled on ice and 20 µL of induced culture and 20 µL of non-induced control culture were boiled with SDS loading buffer at 95°C for 5 min. and then loaded in SDS-PAGE. Ni-NTA purification was performed following manufacturer instructions (QiaExpressionist - Qiagen) and using E. coli BL21(DE3) bacterial cells and NI-NTA resin. Briefly, after IPTG induction of protein expression and culture centrifugation, bacterial cells were resuspended with Denaturing Binding Buffer (Insoluble Fraction – IF) and filter sterilized. Two mL of Ni-NTA resin was added to IF and left in agitation overnight at R.T. and then the resin was packed in one empty column. The resin was washed with 50 volumes (100 mL) of Denaturing Washing Buffer and the bound fraction was eluted twice with 2 mL of Denaturing Elution Buffer (dEB) pH = 5.9 and other 7 times with dEB pH = 4.5. All fractions were immediately neutralized to pH 7.4. The eluted fractions were loaded on SDS-PAGE to estimate the purity of recombinant OmpK36.

### ELISA with Fabs and Purified OMPs

Eight **µ**g of each OMP were coated on a 96 well ELISA plate in PBS, pH = 5.0 overnight at 4°C. The following day, the plate was blocked with PBS/BSA1%, and purified Fabs or freeze-and-thaw preparations were added (about 8 µg/ml) to each well. After washing with PBS/0.1% Tween-20, the anti-Human-Fab antibody-HRP (SIGMA-Aldrich), was added and incubated for 45–60 min at 37°C. After washing with PBS/0.1% Tween-20, 40 **µ**L of TMB solution (Pierce) was added and the plate read at 450 nm.

### Western Blot on Induced/Non-Induced *E. coli BL21(DE3)* Total Lysates with Purified Fabs

Western Blot (WB) was performed on total bacteria proteins from IPTG induced or non-induced cultures or on purified OmpK36 or OmpF proteins. Protein concentration was determined by BCA kit (Pierce), following manufacturer instructions. Five hundred ng of purified protein or 20 **µ**L of total cell lysate was loaded in each well. Proteins were transferred to PVDF membrane at 0.350 A for 2 hours, then the membrane was blocked with PBS/10% BSA at 4°C in agitation overnight. The day after the membrane was incubated with Fab7816 (10 **µ**g/mL) diluted in PBS/BSA5% for 1 hour at R.T. and then washed 3 times with PBS/0.1% Tween-20. The membrane was incubated for 1 hour at R.T. with HRP-conjugated anti-Human Light Chain K antibody (Pirce) and developed by SuperSignal West Pico Chemiluminescent Substrate (Pierce), following manufacturer instructions. WB was also developed with anti-HIS-HRP antibody (Roche) to verify the presence of HIS-tag at N-term of OmpK36.

### Bidimensional Electrophoresis (2DE) and Mass Spectrometry on Carotid Plaque Lysates

A carotid plaque fragment was weighted (65 mg), cut in small fragments and directly smashed with a Dounce in 500 **µ**L of R5 buffer, containing: 8 M urea, 2M thiourea, 4% CHAPS, 0.05% Zwittergent, 40 mM Trizma base and a cocktail of protease inhibitors. The lysate was then sonicated for 5 min and centrifuged at 13,000 rpm for 30 min at 15°C. Supernatant protein content was evaluated by BCA (Pierce). The proteins (200 **µ**g) were dissolved in R5 buffer (final volume 130 **µ**L), and then added to DeStreak (100 mM) and 2% IPG buffer pH 3-10NL, prior to loading the sample on 7 cm strip pH 3-10NL. Total focusing run for the 1st dimension was 50,000 Vh The 2nd dimension was performed using 12.5% acrylamide SDS-PAGE.

For the 2D electrophoresis Western Blotting (2DE-WB), the proteins (70 **µ**g) dissolved in R5 buffer (final volume 130 **µ**l), were added of DeStreak (100 mM) and 2% IPG buffer pH 3-10NL, prior to loading the sample on strip pH 3-10NL, 7 cm. After 2D electrophoresis proteins were then transferred to PVDF membrane by semi-dry electroblotting and probed with Fab 7816-FLAG. Immune complexes were visualized by incubation with HRP-conjugated anti-FLAG antibody (Sigma-Aldrich) and chemiluminescent detection.

Protein spots of interest were excised from the gel and digested with trypsin and directly analysed by MALDI-ToF mass spectrometry, using alpha-cyano-4-hydroxycinnamic acid (Sigma-Aldrich) as matrix. Mascot software (Matrix Science) was used for protein searching in IPI_human_20100623 database. Identification was accepted when the Mascot score was >66, with a good sequence coverage.

### Western Blot with Preparation of Purified Human TAGLN

The binding of representative Fabs248, Fab172, Fab1630 and Fab7816 to TAGLN was performed using 400 ng of purified TAGLN (Origene, TP302448) Myc-tagged loaded in each lane. TAGLN was run in SDS-PAGE and then transferred on PVDF membrane for 2 h at 0.350 A. PVDF was then blocked with PBS/10% BSA or PBS/10% Milk O/N at 4°C in agitation. Anti-Myc-Peroxidase (Abcam, ab62928 in PBS/BSA5%) and anti-human TAGLN (Abnova, H00006876-M01 in PBS/5% Milk) commercial antibodies were used as positive controls. Purified Fabs were used in the assay. Anti-Light Chain K-Peroxidase antibody (PSB/5% BSA) and anti-Mouse-Fab antibody (PBS/Milk 5%) were used to reveal Fab binding or commercial anti-TAGLN binding respectively. After extensive washing with PBS/Tween-20 0.1% WB was developed with SuperSignal West Pico Chemiluminescent Substrate (Pierce).

### Histology, Immunohistochemistry and Immunofluorescence on Plaque Sections

Histology and immunofluorescence/immunohistochemistry were performexd on two coronary plaques and 31 carotid plaques harvested during endoarterectomy immediately after excision. Specimens were fixed in 4% paraformaldehyde in PBS. Carotid plaques were cut in consecutive 2 mm thick rings, decalcified in Osteodec (BioOptica) when required. All biopsies were cryoprotected in 10% sucrose in PBS, embedded in killik (BioOptica), snap frozen in isopentane/liquid nitrogen and stored at −80°C. Cryosections (5** µ**m thick) were obtained from every ring/sample by Leica CM1850 cryostat (Leica Microsystems GmbH) and stained either with Hematoxylin/Eosin or Movat’s pentachrome. The morphology was examined in 4 sections/ring by light microscope Eclipse 55i equipped with DS-L1 camera, and multiple images were composed by LUCIA 4.82 software (Nikon) to reconstruct the section. Carotid plaques were classified as stable, vulnerable or unstable, following Virmani’s modified AHA classification [44]. Serial sections (10 µm thick) representative of the lesion or without atheroma were selected for each specimen and submitted to immunolabeling. To avoid unspecific reactivity with immune complexes or endogenous human IgG in human atherosclerotic lesions, Fab7816 was tagged with a recombinant artificial FLAG tag, as previously described [45]. Different combinations of antibodies were tested in single or multiple labelling. The following antibodies were used: Fab7816-FLAG (100 ng/mL, overnight, 4°C) detected either with anti-FLAG-FITC (diluted 1∶100 1 h R.T., clone M2, Sigma-Aldrich), or mouse-anti-FLAG–HRP (diluted 1∶100 1 h R.T., clone M2, Sigma-Aldrich) revealed by DAB detection kit (*Vector* Laboratories Inc); mouse-anti-TAGLN (clone H00006876-M01) and goat anti- human TAGLN polyclonal antibody (diluted 1∶200 and 1∶100 respectively, overnight 4°C, both from Abnova Corporation) revealed either by goat-anti-mouse IgG(H+L) AlexaFluor594 or goat-anti-mouse IgG2a AlexaFluor488, or donkey-anti-goat IgG AlexaFluor488, or donkey-anti-goat IgGAlexaFluor680 (diluted 1∶500 45 min RT, all from Molecular Probes Invitrogen); mouse-anti-human CD68 (diluted 1∶100 2 h RT, clone KP-1, DAKO) revealed by goat-anti-mouse IgG1k AlexaFluor 488 or goat-anti-mouse IgG(H+L) AlexaFluor594; mouse-anti-human Collagen type I (diluted 1∶100, overnight at 4°C, Calbiochem Merck, Darmstadt, Germany) revealed by goat-anti-mouse IgG AlexaFluor680 or goat-anti-mouse IgG AlexaFluor488; mouse-anti-human CD45-PE (diluted 1∶100, 1 h RT, BD Biosciences, Franklin Lakes, NJ USA). Unrelated E8Fab-FLAG [25], [45] or omission of the primary antibodies served as negative controls. Single staining was performed in parallel to double or multiple as positive controls. DAPI (Sigma-Aldrich) or Mayer’s Haematoxylin opportunely counterstained the nuclei. Sections were examined under Nikon Eclipse 55i microscope or under Leica TCS SP5 confocal microscope (Leica Microsystems). Two scientists evaluated the sections independently, and transmitted light and fluorescence images were obtained. Confocal 2D free projection max from Z-series of images were acquired in single or double channel, respectively, then superposed by Leica LAS AF software and mounted in panels by AdobePhotoshop CS.

Generation of fibrocytes in vitro. Buffy Coats from healthy donors were collected as previously described [46] and peripheral blood mononuclear cells (PBMCs) were isolated by density-gradient centrifugation using Histopaque-1077) according to the manufacturer’s instructions. CD14+ monocytes were isolated by positive selection with magnetic MicrobBeads (Miltenyi Biotec, Bisley, UK). Fibrocytes were generated by culture in serum-free growth medium (SFM, RPMI 1640, Life Technologies, supplemented with 40 U/mL penicillin and 0.4 mg/mL streptomycin, 1%HEPES, 1% liquid media supplement ITS+3, 1% non-essential amino acids, 1% Sodium Pyruvate). CD14+ cells (2,5×10^5^/mL) were resuspended in SFM and cultured on 13mm diameter round glass coverslips into 24-well plastic plates in a humidified incubator at 37°C, 5% CO_2_ for 4 days. MRC-5 cells (human fetal lung fibroblasts**,** CCL-171™, ATCC) at passage 25 were similarly seeded at density of 2,5×10^5^/mL in MEM, Life Technologies, supplemented with 10% FBS and 40 U/mL penicillin and 0.4 mg/mL streptomycin to serve as controls. Reagents were provided by SIGMA-Aldrich, Steinheim, Germany whereas not indicated.

### Immunofluorescence on Fibrocytes

CD14^+^ fibrocytes and MRC-5 cells freshly-fixed in 2% paraformaldehyde in PBS were rinsed in PBS, permeabilized in 0.1% TritonX100 in PBS, then submitted to immunofluorescence against 7816FabFLAG, E8FabFLAG, CD45, CD68, Collagen Type I and TAGLN similarly to plaque sections. Specimens were analyzed at confocal microscope as described above.

## Supporting Information

Figure S1 **Immunofluorescence and mmunohistochemistry on human atherosclerotic carotid and coronary plaques with 7816Fab-FLAG and not-correlated E8Fab-FLAG.** Representative carotid plaque serial sections (A–C) display the presence of signal (brown) with 7816Fab-FLAG (A) in several cells of the vessel wall by Immunoperoxidase, but its absence either with its omission (B) or substitution with a not correlated antibody, the E8Fab-FLAG (C). Haematoxylin stains nuclei (blue). Asterisk indicates the vascular lumen, scale bar the magnification. Confocal microscopy on coronary plaque sections display specific binding of 7816Fab-FLAG (D) but not of the Fab E8Fab-FLAG (E). DAPI stains the nuclei (blue).
(TIF)

Figure S2 **Histology and Confocal microscopy on human atherosclerotic carotid.** Figure S2. Histology and Confocal microscopy on human atherosclerotic carotid Haematoxylin & eosin (panel A, upper image) and Movat^1^s (panel A, bottom image) stains of the carotid plaque displayed in B–I confocal microscopy images. 7816Fab-FLAG+ localized the positive cells nearby the lumen (A, B, D asterisks) and in regions rich in both foam cells and SMC. Double labelling (B–F) of a representative field is shown in serial sections stained with 7816Fab-FLAG (green) (B,C, D, FG,I), and either with goat-anti-human TAGLN((white, C), or mouse-anti-human CD68 (red, E, F, H, I). In panels B,C the region with 7816Fab-FLAG+ cells is enlarged in the bottom images, while G, H, I magnified details of the positive region in D, E, F respectively. DAPI stains the nuclei (blue). 7816Fab-FLAG and the other two markers are acquired in single channel to avoid crosstalk signals, then electronically merged by Leica LCS-Lite software. Scale bars indicate the magnification.
(TIF)

Figure S3 **Immunohistochemistry on human atherosclerotic carotid with 7816Fab-FLAG and immunofluorescence with monoclonal anti-TAGLN.** Immunoperoxidase shows a region of the vessel wall (A) with 7816Fab-FLAG+ cells (brown) (B), corresponding by immunofluorescence on serial sections to an area rich in TAGLN smooth muscle cells (green) (C). Either Haematoxylin or DAPI stains the nuclei (blue). Scale bars indicate the magnification.
(TIF)

Figure S4 **Confocal microscopy with 7816Fab-FLAG on human atherosclerotic carotids: negative controls.** A representative field from a plaque, which failed to display any immunoreactivity with 7816Fab-FLAG (A–C) stained with multiple labelling vs. 7816Fab-FLAG, TAGLN and CD68 is shown. Negative control without any of the primary antibodies, but all the secondary antibodies applied for the multiple staining (D ) demonstrated the absence of specific signal in a serial section of the plaque shown in figure 5 and 6.
(TIF)

Figure S5 **Confocal microscopy with 7816Fab-FLAG on human atherosclerotic carotid plaque and on CD14+ fibrocytes: fibrocyte markers.** Spindle and elongated CD14+ cells cultured for 4 days in the absence of serum express CD45/CD68 (A), CD45/Collagen type I/TAGLN (b), showing a fibrocyte phenotype. DAPI stains nuclei, scale bars indicate the magnification.
(TIF)

Figure S6 **Reactivity of representative Fabs from all patients with TAGLN in ELISA.** ELISA with all representative Fabs on human transgelin. Reactivity against bovine serum albumin (BSA) used as blocking antigen, is also shown. The reconstructed full IgG of Fab7816 was also tested (IgG7816). Two unrelated negative Fabs were used as negative controls.
(TIFF)

Table S1 **Clinical characteristics of patients from which coronary samples were obtained.** NSTEMI: non-ST segment elevation myocardial infarction; UA =  unstable angina with negative troponin; LVEF: left ventricular ejection fraction. 1-D one vessel disease, 2-D =  two vessels disease, 3-D =  three vessels disease. Cx =  circumflex artery, OM =  obtuse marginal artery, LAD  =  left descending artery. QCA  =  quantitative coronary assessment.
(DOC)

Table S2 **Combinatorial phage-display Fab libraries characteristics.** Library extension and sequence analyses of randomly sampled clones. The most represented IGHV or IGKV genes is also shown. The average percentage divergence from germline sequences for each HC and LC were defined on the basis of nucleotide changes in the IGHV or IGKV sequences and the average CDR3 length is described. HC =  heavy chain, LC =  light chain.
(DOC)

Table S3 **Histological, clinical and functional carotid plaque features.**
(DOC)

Protocol S1 **Expression and purification of IgG7816 by baculovirus in insect cells.**
(DOC)

Protocol S2 **Germline reversion of Fab 7816.**
(DOC)
