# Supplementary figures and images for: Cross-Reacting Antibacterial Auto-Antibodies Are Produced within Coronary Atherosclerotic Plaques of Acute Coronary Syndrome Patients
Source: PLoS One. 2012 Aug 6;7(8):e42283. doi: 10.1371/journal.pone.0042283 (PMC3412836; doi:10.1371/journal.pone.0042283)

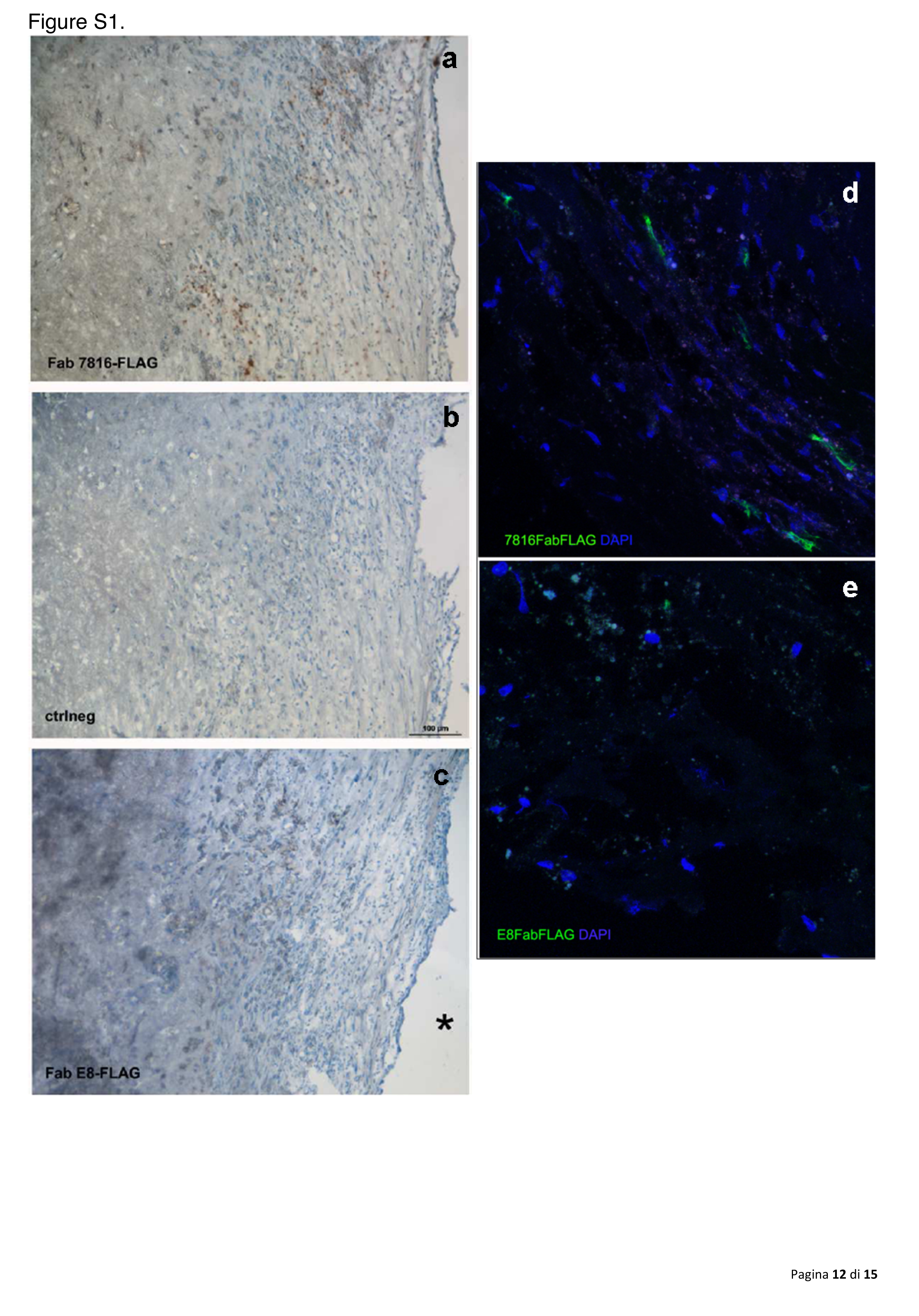

Supplement: Figure S1 — Immunofluorescence and mmunohistochemistry on human atherosclerotic carotid and coronary plaques with 7816Fab-FLAG and not-correlated E8Fab-FLAG. Representative carotid plaque serial sections (A–C) display the presence of signal (brown) with 7816Fab-FLAG (A) in several cells of the vessel wall by Immunoperoxidase, but its absence either with its omission (B) or substitution with a not correlated antibody, the E8Fab-FLAG (C). Haematoxylin stains nuclei (blue). Asterisk indicates the vascular lumen, scale bar the magnification. Confocal microscopy on coronary plaque sections display specific binding of 7816Fab-FLAG (D) but not of the Fab E8Fab-FLAG (E). DAPI stains the nuclei (blue). (TIF) [file pone.0042283.s001.tif]

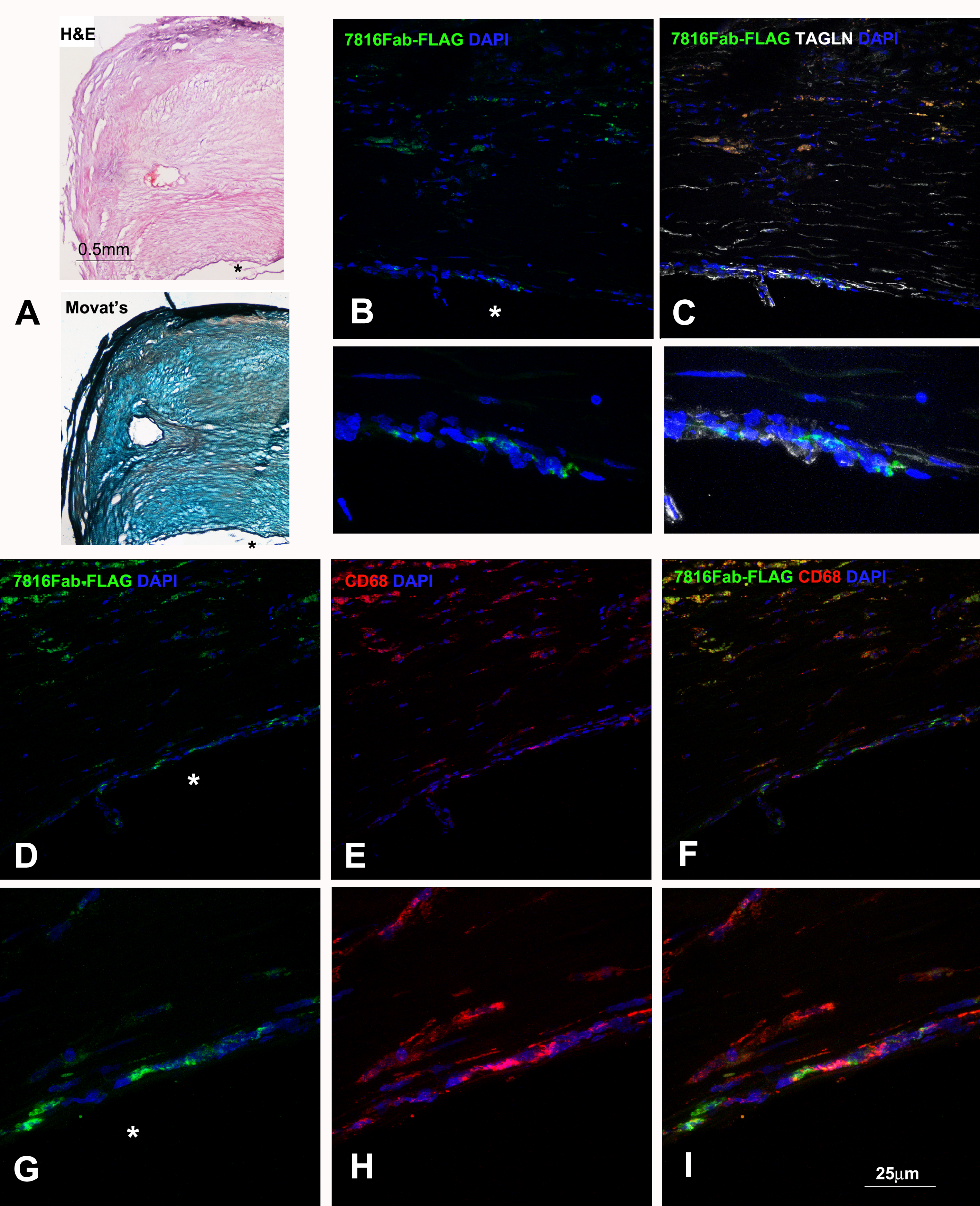

Supplement: Figure S2 — Histology and Confocal microscopy on human atherosclerotic carotid. Figure S2. Histology and Confocal microscopy on human atherosclerotic carotid Haematoxylin & eosin (panel A, upper image) and Movat1s (panel A, bottom image) stains of the carotid plaque displayed in B–I confocal microscopy images. 7816Fab-FLAG+ localized the positive cells nearby the lumen (A, B, D asterisks) and in regions rich in both foam cells and SMC. Double labelling (B–F) of a representative field is shown in serial sections stained with 7816Fab-FLAG (green) (B,C, D, FG,I), and either with goat-anti-human TAGLN((white, C), or mouse-anti-human CD68 (red, E, F, H, I). In panels B,C the region with 7816Fab-FLAG+ cells is enlarged in the bottom images, while G, H, I magnified details of the positive region in D, E, F respectively. DAPI stains the nuclei (blue). 7816Fab-FLAG and the other two markers are acquired in single channel to avoid crosstalk signals, then electronically merged by Leica LCS-Lite software. Scale bars indicate the magnification. (TIF) [file pone.0042283.s002.tif]

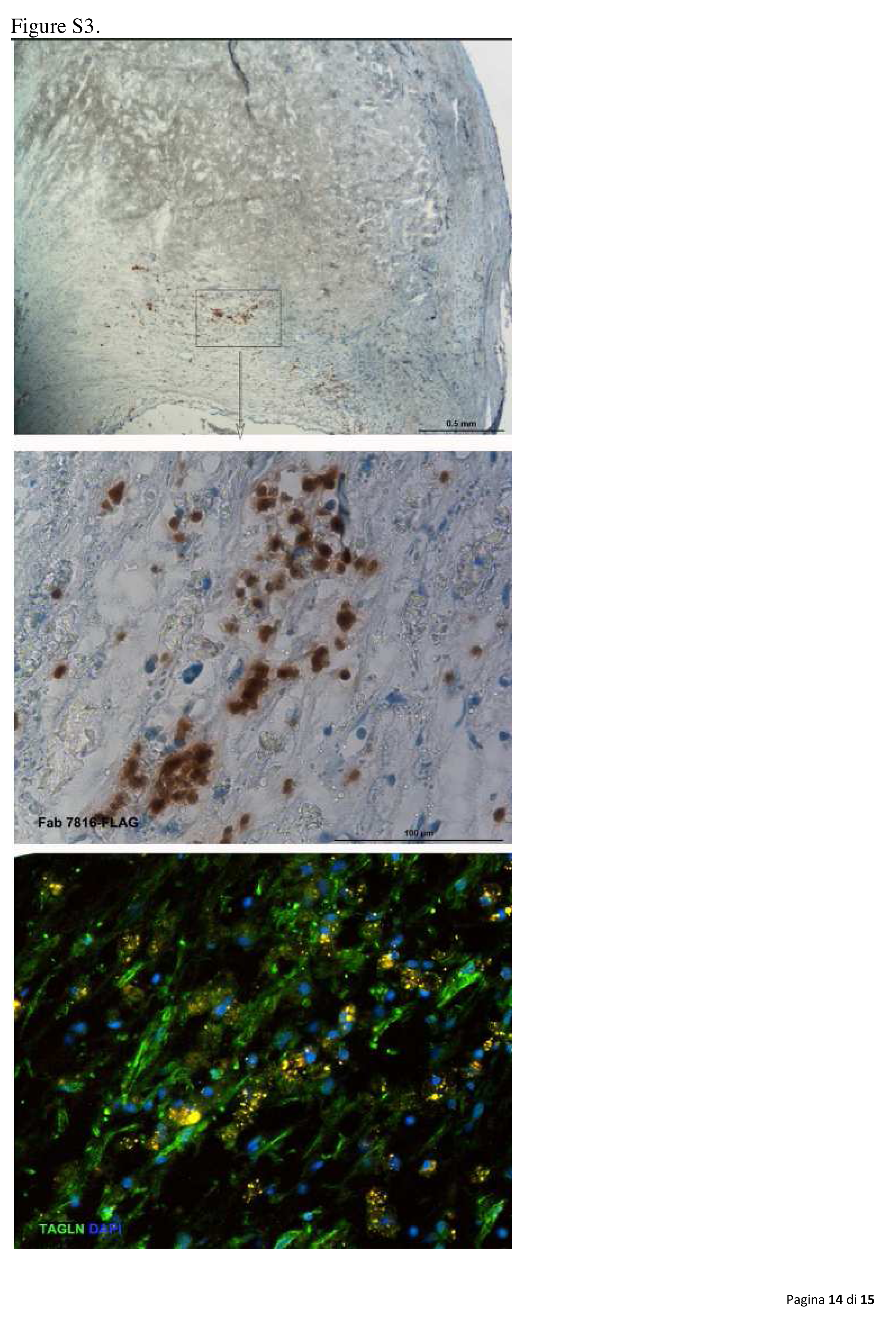

Supplement: Figure S3 — Immunohistochemistry on human atherosclerotic carotid with 7816Fab-FLAG and immunofluorescence with monoclonal anti-TAGLN. Immunoperoxidase shows a region of the vessel wall (A) with 7816Fab-FLAG+ cells (brown) (B), corresponding by immunofluorescence on serial sections to an area rich in TAGLN smooth muscle cells (green) (C). Either Haematoxylin or DAPI stains the nuclei (blue). Scale bars indicate the magnification. (TIF) [file pone.0042283.s003.tif]

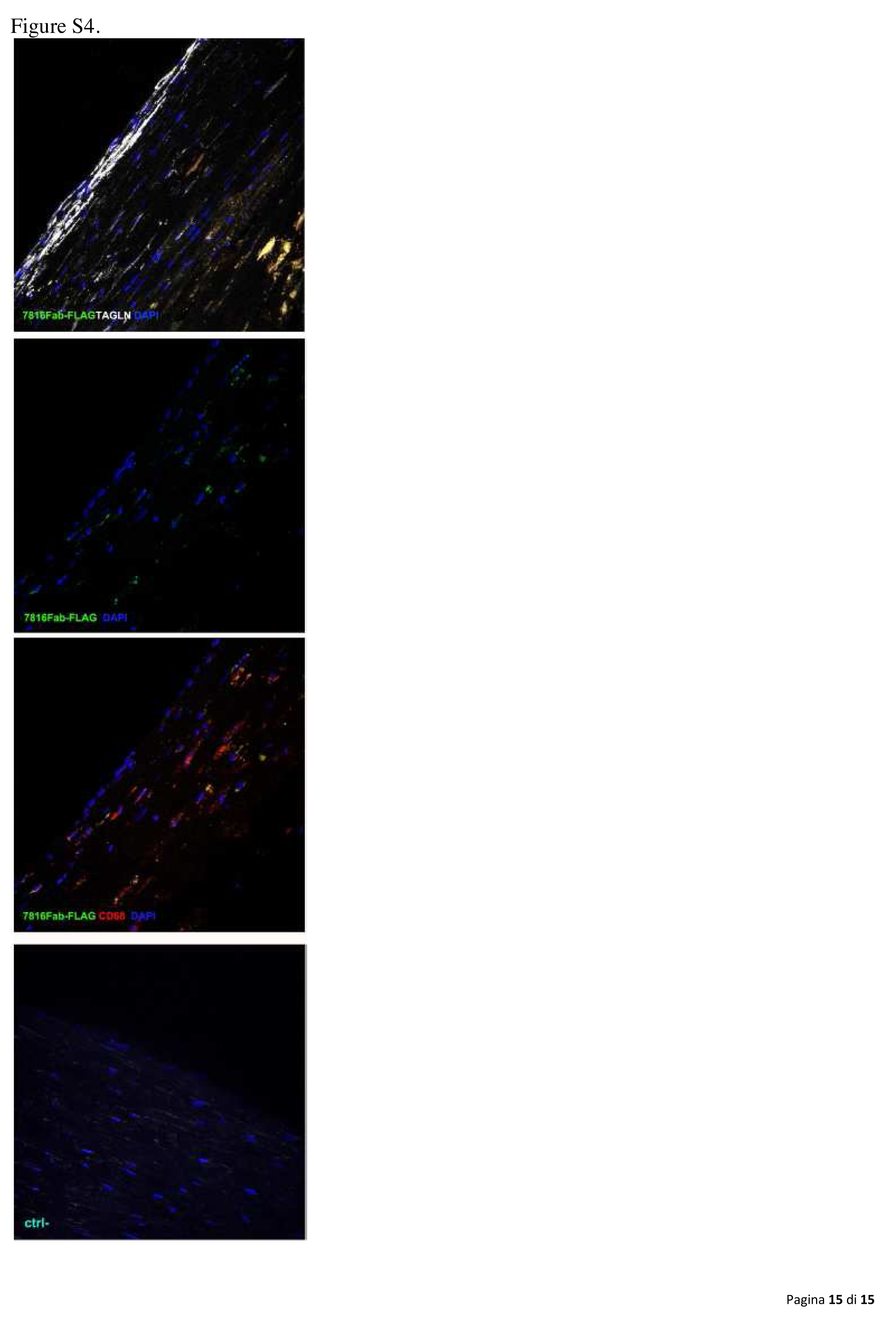

Supplement: Figure S4 — Confocal microscopy with 7816Fab-FLAG on human atherosclerotic carotids: negative controls. A representative field from a plaque, which failed to display any immunoreactivity with 7816Fab-FLAG (A–C) stained with multiple labelling vs. 7816Fab-FLAG, TAGLN and CD68 is shown. Negative control without any of the primary antibodies, but all the secondary antibodies applied for the multiple staining (D ) demonstrated the absence of specific signal in a serial section of the plaque shown in figure 5 and 6. (TIF) [file pone.0042283.s004.tif]

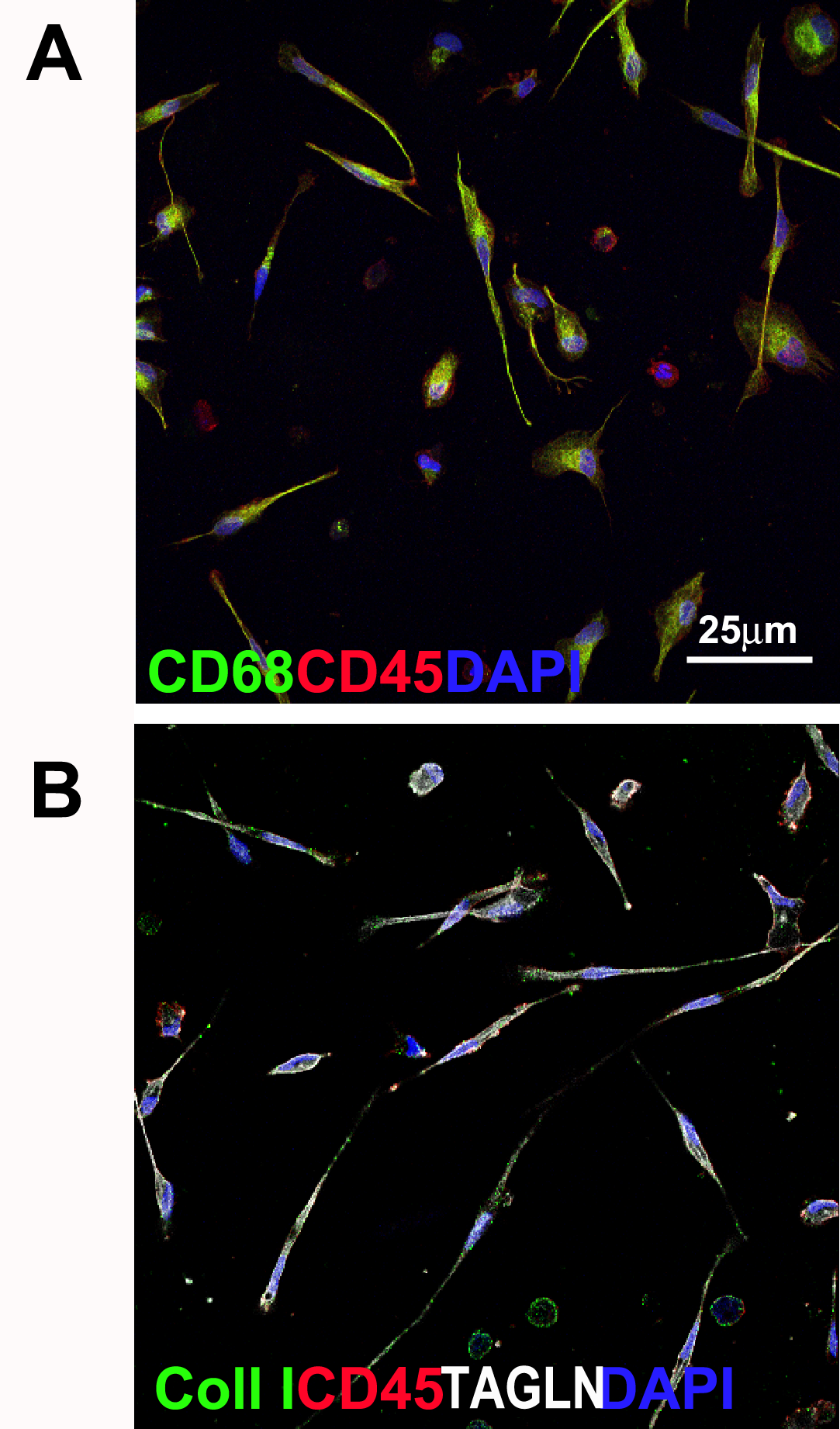

Supplement: Figure S5 — Confocal microscopy with 7816Fab-FLAG on human atherosclerotic carotid plaque and on CD14+ fibrocytes: fibrocyte markers. Spindle and elongated CD14+ cells cultured for 4 days in the absence of serum express CD45/CD68 (A), CD45/Collagen type I/TAGLN (b), showing a fibrocyte phenotype. DAPI stains nuclei, scale bars indicate the magnification. (TIF) [file pone.0042283.s005.tif]

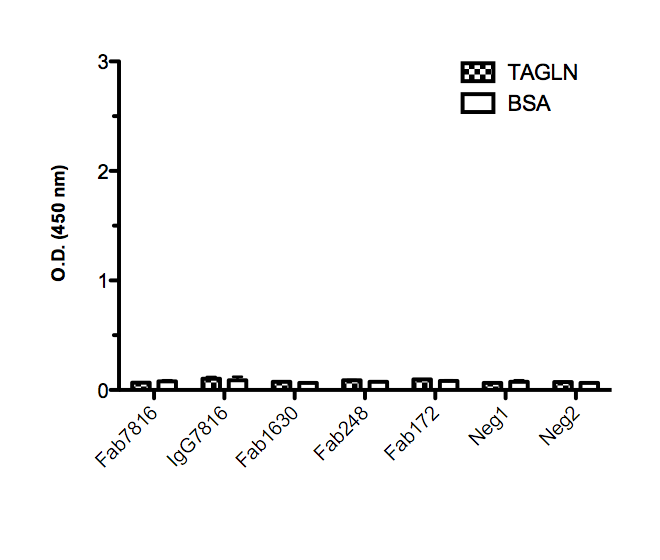

Supplement: Figure S6 — Reactivity of representative Fabs from all patients with TAGLN in ELISA. ELISA with all representative Fabs on human transgelin. Reactivity against bovine serum albumin (BSA) used as blocking antigen, is also shown. The reconstructed full IgG of Fab7816 was also tested (IgG7816). Two unrelated negative Fabs were used as negative controls. (TIFF) [file pone.0042283.s006.tiff]
