# Supplementary material for: Cross-Reacting Antibacterial Auto-Antibodies Are Produced within Coronary Atherosclerotic Plaques of Acute Coronary Syndrome Patients
Source: PLoS One. 2012 Aug 6;7(8):e42283. doi: 10.1371/journal.pone.0042283 (PMC3412836; doi:10.1371/journal.pone.0042283)
Supplement: Table S1 — Clinical characteristics of patients from which coronary samples were obtained. NSTEMI: non-ST segment elevation myocardial infarction; UA = unstable angina with negative troponin; LVEF: left ventricular ejection fraction. 1-D one vessel disease, 2-D = two vessels disease, 3-D = three vessels disease. Cx = circumflex artery, OM = obtuse marginal artery, LAD = left descending artery. QCA = quantitative coronary assessment. (DOC) [file pone.0042283.s007.doc]

| **Plaque ID** | **Age** | **Family History** | **Hypertension** | **Cholesterol** | **Smoking** | **Vessels** | **Plaque** | **Clinical presentation** | **LVEF,%** | **QCA** |
| --- | --- | --- | --- | --- | --- | --- | --- | --- | --- | --- |
|  |  |  |  |  |  |  | **localization** |  |  | **(%stenosis)** |
| **ID-A** | 47 | NO | YES | YES | YES | 2-D | LAD | UA | 56 | 88% |
| **ID-B** | 59 | YES | YES | NO | EX | 1-D | OM | NSTEMI | 55 | 59% |
| **ID-C** | 68 | YES | NO | NO | EX | 1-D | Cx | NSTEMI | 45 | 68% |
| **ID-D** | 29 | NO | NO | NO | NO | 1-D | LAD | NSTEMI | 55 | 56% |
