# Supplementary material for: Cross-Reacting Antibacterial Auto-Antibodies Are Produced within Coronary Atherosclerotic Plaques of Acute Coronary Syndrome Patients
Source: PLoS One. 2012 Aug 6;7(8):e42283. doi: 10.1371/journal.pone.0042283 (PMC3412836; doi:10.1371/journal.pone.0042283)
Supplement: Table S2 — Combinatorial phage-display Fab libraries characteristics. Library extension and sequence analyses of randomly sampled clones. The most represented IGHV or IGKV genes is also shown. The average percentage divergence from germline sequences for each HC and LC were defined on the basis of nucleotide changes in the IGHV or IGKV sequences and the average CDR3 length is described. HC = heavy chain, LC = light chain. (DOC) [file pone.0042283.s008.doc]

| **Patient** | **Library extension** | **Ig Chain** | **Nr. Sequenced Clones**  **(clones with**  **different junctions)** | **Most Represented**  **V-Gene**  **(Nr. different clones)** | **V-Gene Avg. Homology** | **CDR3 Length**  **Avg. (aa)** |
| --- | --- | --- | --- | --- | --- | --- |
| **ID-A** | ***1 x 10^6^*** | HC | 53 (19) | V_H_4-61 | 91,00 | 15,33 |
|  |  | LC | 50 (21) | Vκ3-20 | 94,17 | 9,50 |
| **ID-B** | ***5 x 10^5^*** | HC | 39 (19) | V_H_3-23 | 92,81 | 10 |
|  |  | LC | 36 (6) | Vκ3-15 | 95,30 | 10,00 |
| **ID-C** | ***1x10^5^*** | HC | 44 (5) | V_H_3-23 | 92,52 | 16,66 |
|  |  | LC | 39 (16) | Vκ1-39 | 96,04 | 8,67 |
| **ID-D** | ***1.5x10^6^*** | HC | 46 (15) | V_H_4-4 | 91,56 | 12,36 |
|  |  | LC | 40 (12) | Vκ1-39 | 93,19 | 9,00 |
