# Supplementary material for: Cross-Reacting Antibacterial Auto-Antibodies Are Produced within Coronary Atherosclerotic Plaques of Acute Coronary Syndrome Patients
Source: PLoS One. 2012 Aug 6;7(8):e42283. doi: 10.1371/journal.pone.0042283 (PMC3412836; doi:10.1371/journal.pone.0042283)
Supplement: Table S3 — Histological, clinical and functional carotid plaque features. (DOC) [file pone.0042283.s009.doc]

| **Histology**  **Plaque Features** | **n / n_tot_** | **n_7816+_ / n_tot_** |
| --- | --- | --- |
| **necrotic core** | 24/31 | 14/19 |
| **thin cup** | 20/29 | 11/19 |
| **calcification** | 27/31 | 16/19 |
| **fibrolipids** | 31/31 | 19/19 |
| **inflammatory infiltrate** | 23/31 | 13/19 |
| **thrombosis/haemorrage** | 14/31 | 8/19 |
|  |  |  |
| **Instability** | 16/31 | 9/19 |
|  |  |  |
| **Eco-Doppler**  **Plaque Features** |  |  |
| **Stenosis >70%** | 16/31 | 10/19 |
| **Instability** | 12/31 | 6/19 |
