# Supplementary material for: Cross-Reacting Antibacterial Auto-Antibodies Are Produced within Coronary Atherosclerotic Plaques of Acute Coronary Syndrome Patients
Source: PLoS One. 2012 Aug 6;7(8):e42283. doi: 10.1371/journal.pone.0042283 (PMC3412836; doi:10.1371/journal.pone.0042283)
Supplement: Protocol S1 — Expression and purification of IgG7816 by baculovirus in insect cells. (DOC) [file pone.0042283.s010.doc]

**Protocol S1. Expression and purification of IgG7816 by baculovirus in insect cells**. Heavy and light chains of Fab7816 were cloned in pAc-k-Fc vector (PROGEN) to allow the production of IgG7816 in Sf9 (Invitrogen) insect cells, following manufacturer instruction. Sf9 cells were infected with a 0.2 MOI and grown for 5 days at 27°C in agitation. At day 5, the cell culture was centrifuged at 2000 RPM for 15 min, the supernatant was harvested, filtered and PMSF was added. The supernatant was loaded on a chromatographic column with protein G resin. The resin was washed with 100-150 mL of PBS pH=7.4 (F.R.: 1-2 mL/min), and the bound IgG was then eluted with 10 mL of citric acid 0.1 M pH=5.2 and the solution neutralized at pH=7.0 with 1.2 mL of Tris 2M pH=11.
