# Supplementary material for: Cross-Reacting Antibacterial Auto-Antibodies Are Produced within Coronary Atherosclerotic Plaques of Acute Coronary Syndrome Patients
Source: PLoS One. 2012 Aug 6;7(8):e42283. doi: 10.1371/journal.pone.0042283 (PMC3412836; doi:10.1371/journal.pone.0042283)
Supplement: Protocol S2 — Germline reversion of Fab 7816. (DOC) [file pone.0042283.s011.doc]

**Protocol S2. Germline reversion of Fab 7816.** A germline light chain with the same combination of minigenes VK, JK and the same junction of light chain of Fab7816 was found in peripheral blood of one patient. Germline reversion of the heavy chain was carried out by overlapping PCRs using three pairs of specific primers annealing respectively on the FR2, FR3 and the CDR3 regions (78VHFW AAACTGCTCGAGTCGGGCCCAGGACTGGTGAAGCCT and 78FR2RW GTAATTGGTGCTCCCACTGGTATAGATTCGCCCAATCCA; 78FR2FW GGTTCTTACTACTGGAGCTGGATCCGGCAGCCCGCCGGG; and 78FR3RW GCTCAGTTTCAGGGAGAACTGGTTCTTGGACGTGTCCAC; and 78FR3FW ACCAATTACAACCCCTCCCTCAAGAGTCGAGTCACCATAT and CH1RW; 78FR3AFW CTGAGCTCTGTGACCGCCGCAGACACGGCCGTGTATTACTG and CG1Z. The complete heavy chain was then amplified by using Vh1f (CAGGTGCAGCTGCTCGAGTCTGGG) and CG1z primers.
